# Evaluation of ventricular systolic function by speckle tracking technique in patients with biliary atresia before and after liver transplantation

**DOI:** 10.1038/s41598-021-97096-8

**Published:** 2021-09-08

**Authors:** Márcio Miranda Brito, Helena Thie Miyatani, Paulo Renato de Alencar Pereira, Ana Cristina Aoun Tannuri, Uenis Tannuri

**Affiliations:** 1 Heart Institute (InCor), University of São Paulo Medical School, Avenida Dr. Enéas Carvalho de Aguiar, 44, São Paulo, Cerqueira CesarSP 05403-900 Brazil; 2 Division of Pediatric Surgery and Liver Transplantation Unit, University of Sao Paulo Medical School, Sao Paulo, SP Brazil

**Keywords:** Cardiology, Medical research

## Abstract

To evaluate the ventricular function of patients with biliary atresia (BA) before and after liver transplantation using two-dimensional speckle tracking. Observational, analytical study with healthy control group, volunteers. We recruited patients from 0 to 18 years old who were candidates for liver transplantation and patients after six months of liver transplantation performed for BA from January 1997 to August 2015 at Children’s Institute of São Paulo University Medical School. The patients were submitted to a complete conventional echocardiographic study. After that, the images were captured for global longitudinal strain (GLS). A blood sample was collected for brain natriuretic peptide (BNP) level. Ejection fraction obtained by Simpson’s method was significantly higher in the hepatic pre-transplantation group (p < 0.001), as well as left atrial size (p < 0.001) and left ventricle size (p = 0.039). The left ventricular mass index was significantly higher in pre-transplantation group (p < 0.001). The left atrium volume (p = 0.008) and the left ventricular mass index (p t = 0.035) were higher in the post-transplant group. It was observed that the lower the BNP, the lower/more negative the GLS in the post-transplant group (p = 0.038 and r = 0.427). Significant reduction in the overall longitudinal *strain* of the left ventricle was detected before (p = 0.01) and after liver transplantation (p = 0.019). A subclinical left ventricular systolic dysfunction was evidenced by two-dimensional speckle tracking technique before and after liver transplantation, even when compared to normal values of the last pediatric meta-analysis.

## Introduction

Biliary atresia (BA) is an obstructive cholangiopathy and the main cause of neonatal cholestasis. It progresses fast to secondary biliary cirrhosis, leading to death in the first two years of life when untreated^1^. Currently, BA is the most frequent indication for liver transplantation in most pediatric centers.

Cirrhotic cardiomyopathy, initially described in 1989, is defined by the following criteria: normal or increased systolic left ventricular contractility at rest; attenuated systolic contraction or diastolic relaxation in the presence of pharmacological, physiological or surgical stress, cardiac electrical abnormalities and myocardial hypertrophy.^1^ A working group meeting at the World Congress of Gastroenterology in 2005, elaborated a proposal for the definition of cirrhotic myocardiopathy and described a serie of abnormalities to the resting Doppler echocardiogram in adults: E/A ratio < 1,: E-wave deceleration time > 200 ms, isovolumetric relaxation time > 80 ms, left atrial volume increase, decreased left ventricular contractility, hypokinesis and / or wall akinesia, increased myocardial mass, lower ejection fraction (< 55%), increased brain natriuretic peptide and troponin levels^2^. The diagnostic criteria for children are not well established or validated.

According to current recommendations, echocardiography is the first-line imaging method in the evaluation of patients with suspected cirrhotic cardiomyopathy, providing structural and functional information. However, many patients may have preserved resting systolic function assessed by conventional techniques. New echocardiographic methods may overcome some of these limitations, allowing a quantitative analysis of the myocardial deformation^3,4^. The main objective of this study is to evaluate the ventricular function of patients with biliary atresia before and after pediatric liver transplantation using new echocardiographic techniques such as two-dimensional speckle tracking. The deformation analysis by speckle tracking is based on the tracking of natural acoustic markers present in the two-dimensional gray scale image. The speckles function as a “fingerprint” of that determined myocardial segment and it is possible to calculate the deformation and the speed with which the deformation occurs from the displacement of these markers.

## Patients and methods

### Patients

This is an observational, analytical, cross-sectional study with a control group, which included patients from zero to eighteen years old who were candidates for liver transplantation due to BA with severe cirrhosis, patients from zero to eighteen years admitted to liver transplantation due to BA from 1989 to 2015 at Children’s Institute of University of São Paulo Medical School and healthy volunteers controls of primary care units paired by sex and age. From July 2016 to May 2018, 120 patients were included. The selected children were divided into four groups, each one with 30 participants: patients who were candidates for liver transplantation, healthy controls in the pre-transplant group, patients post-liver transplantation who had any of the following altered parameters on hepatic pre-transplant echocardiography: dilated cardiac chambers, ventricular hypertrophy, systolic dysfunction, diastolic alteration; and healthy controls in the post-transplant group. Of the thirty patients who were candidates for liver transplantation, ten patients underwent a Kasai portoenterostomy surgery, with surgery performed at an average of 60 days of life, all with jaundice clearance failure.

The diagnosis of cirrhosis was established by a combination of biochemical, clinical, liver biopsy, and ultrasonographic findings. Patients with congenital and other acquired heart diseases such as structural heart diseases, pericardial effusion, arrhythmia, systemic arterial hypertension, other systemic conditions that could affect cardiac function, hepatic retransplantation, hospitalization in the last month prior to the examination and inadequate echocardiographic image were excluded from the study. Informed consent was obtained from all included patients and controls. The study was approved by the Research Ethics Committee of the Faculty of Medicine of University of São Paulo Medical School. All experiments were performed in accordance with relevant guidelines and regulations.

### Clinical evaluation

The patients were interviewed based on a structured questionnaire containing the date of birth, sex, age at the time of the liver transplantation, PELD (Pediatric End-Stage Liver Disease), current medications, comorbidities such as renal failure and traditional risks for cardiovascular disease such as: positive family history, presence of hypertension, diabetes, obesity, arrhythmias. A detailed cardiovascular physical examination including heart rate measurement and noninvasive blood pressure was performed by means of an oscillometric method after five minutes of rest according to the norms of the 4th Consensus on the diagnosis, evaluation and treatment of hypertension in children and adolescents^5^, as well as anthropometric evaluation (weight, height).

### Echocardiography

Standard transthoracic echocardiography was performed according to the recommendations of the American Society of Echocardiography and included M-mode, two-dimensional imaging, conventional, and tissue Doppler evaluation at the septal, lateral mitral annulus and lateral tricuspid annulus^6,7^. The equipment used was a Vivid E9 GE Vingmed Ultrasound AS, with multifrequency transducers (with frequencies of 4.5–1.5 MHz and 8–2.7 MHz). Cardiac chamber dimensions were obtained using two-dimensional mode, and left ventricle ejection fraction (LV EF) was calculated by Simpson’s method. LV mass (g) was estimated using Devereaux’s formula according to the Penn convention and indexed for height (m) raised to an exponential power of 2.7^8,9^. Evaluation of LV diastolic function included conventional as well as tissue Doppler-based measurements: mitral E and A velocities, E/A ratio, deceleration time (DT), E/E’ ratio, with E’ being the average of values obtained by tissue Doppler at the septal and lateral annulus. Left atrial volume was estimated using the biplane area-length method (Simpson´s method), and values were indexed to the BSA.

The isovolumic relaxation time (IVRT) was measured for both LV and RV lateral walls. Calculation of global myocardial performance index (MPI index) was performed according to the recommendations of the American Society of Echocardiography^6^.

**Figure 1:**
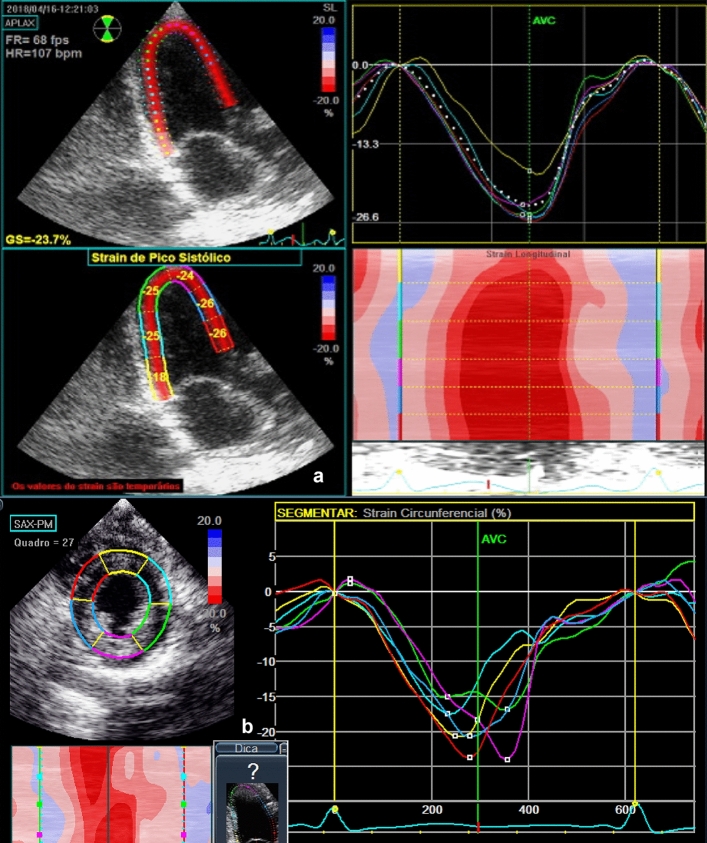
Two-dimensional speckle tracking echocardiogram. (**a**) Apical 3 chamber view, and (**b**) parastenal short axis view at the level of the papillary muscles.

For strain assessment, two-dimensional grey-scale images were acquired in the apical four, three and two-chamber views, with a frame rate of 60–80 fps. In the same way the acquisition of the images in the parasternal window was performed, transverse short axis of the left ventricle in its 03 main cuts: basal, middle and apical. The time "systolic event" was determined by marking the opening and closing moments of the aortic valve. Three cardiac cycles were digitally stored and analyzed using the EchoPAC program BT10 (GE Vingmed Ultrasound AS) (Fig. 1).

### Measurement of brain natriuretic peptide level

A blood sample was collected for the measurement of cardiac biomarkers. The level of BNP assay performed with Triage R (Biosite Diagnostics) test.

### Statistical methods

The software used for the calculations was SPSS 17.0 for windows. The level of significance used for the tests was 5%. For the quantitative variables, this analysis was done by observing the minimum and maximum values, and the calculation of means, standard deviations and median. Absolute and relative frequencies were calculated for the qualitative variables.

For the comparison of means of two groups, Student's t-test was used. In order to test the homogeneity between the proportions, the chi-square test was used. Correlation between variables was assessed using the Pearson correlation coefficient.

### Ethics declaration

The study was approved by the Institutional Ethical Committee.

### Consent to participate

Informed consent was obtained from legal guardians.

## Results

### Demographic and clinical data

Patients in the pre-transplant group and in the control group had similar ages [14.30 (3.00–60.00) vs. 14.33 (3.00–60.00) months; p = 0.994]. There were seventeen female patients in the pre-transplant group (56.7%) and seventeen in the control group (chi-square test, p = 1,000).

Patients from the post-transplant group and from respective control group had similar ages [6.70 (1.00–17.00) vs. 6.70 (1.00–17.00) years; p = 1,000]. There were sixteen (53.3%) female patients in each group (chi-square test, p = 1,000) (Table 1).

**Table 1:** Demographic parameters.

	Pre-Tx (n = 30) Average (SD)	Controls (n = 30) Average (SD)d	P*	Post-Tx (n = 30) Average (SD)d	Controls (n = 30) Average (SD)	P*
Age (months / years)	14.30 (± 15.87)	14.33 (± 15.86)	0.994	6.70 (± 5.47)	6.70 (± 5.47)	1.000
Weight (Kg)	8.62 (± 3.66)	10.25 (± 4.90)	0.151	26.39 (± 17.12)	26.35 (± 18.99)	0.994
Height (cm)	69.73 (± 12.59)	74.93 (± 15.89)	0.166	117.53 (± 31.47)	115.73 (± 33.38)	0.831
Body surfasse area (m^2^)	0.41 (± 0.12)	0.47 (± 0.15)	0.137	0.92 (± 0.40)	0.90 (± 0.44	0.905
Heart rate (bpm)	121.27 (± 17.13)	124.93 (± 15.61)	0.390	98.03 (± 23.49)	91.20 (± 22.01)	0.250
PELD	14.93 (± 9.34)	0	–	18.70 (± 11.19)	0	–
PELD adjusted	44.80 (± 28.03)	0	–	57.63 (± 31.40)	0	–

PELD, Pediatric End-stage Liver Disease; *Students t-test.

The data normality test showed a normal distribution.

### Conventional echocardiogram and tissue Doppler

The ejection fraction obtained by Simpson´s method was significantly lower in the pre-transplant group (60.50 ± 4.49 vs. 66.40 ± 6.56%, p < 0.001) than controls, although it was not below the expected values for normal children. The thickness of the interventricular septum was significantly higher (5.64 ± 1.28 vs. 4.07 ± 0.78 mm, p < 0.001) as well as the posterior wall thickness (5.25 ± 0.96 vs. 3.67 ± 0.71 mm, p < 0.001), the left ventricle diastolic diameter (28.00 ± 4.55 vs. 25.60 ± 4.23 mm, p = 0.039), the systolic diameter of the left ventricle (17, 40 ± 2.95 vs. 15.73 ± 2.88 mm, p = 0.031) and the right ventricle diastolic diameter (14.70 ± 1.97 vs. 13.08 ± 1.98) (Table 2).

**Table 2:** Two-D and M-mode Echocardiogram.

	Pre-Tx (n = 30) Average (SD)	Controls (n = 30) M Average (SD)	P*	Post-Tx (n = 30) Average (SD)	Controls (n = 30) Average (SD)	P*
TAPSE (cm)	1.70 (± 0.26)	1.59 (± 0.29)	0.124	1.86 (± 0.26)	1.90 (± 0.28)	0.536
LV EF Simpson (%)	60.50 (± 4.49)	66.40 (± 6.56)	** < 0.001**	60.72 (± 4.31)	64.07 (± 4.08)	**0.003**
Aortic sinuses (mm)	15.20 (± 2.25)	14.28 (± 2.14)	0.111	21.83 (± 5.04)	20.10 (± 4.21)	0.154
Left Atrium (mm)	22.32 (± 4.03)	15.93 (± 2.39)	** < 0.001**	25.35 (± 4.86)	22.42 (± 4.38)	**0.017**
RVDD (mm)	14.70 (± 1.97)	13.08 (± 1.98)	**0.003**	18.95 (± 3.63)	17.67 (± 3.69)	0.180
LA volume (ml/m^2^)	31.78 (± 6.35)	14.25 (± 2.04)	** < 0.001**	23.24 (± 5.81)	19.66 (± 4.11)	**0.008**
Septum (mm)	5.64 (± 1.28)	4.07 (± 0.78)	**< 0.001**	6.17 (± 1.29)	5.17 (± 1.15)	**0.002**
LV posterior wall (mm)	5.25 (± 0.96)	3.67 (± 0.71)	** < 0.001**	5.96 (± 1.18)	4.77 (± 1.22)	** < 0.001**
LVDD (mm)	28.00 (± 4.55)	25.60 (± 4.23)	**0.039**	36.45 (± 8.07)	36.77 (± 7.09)	0.872
LVSD (mm)	17.40 (± 2.95)	15.73 (± 2.88)	**0.031**	23.17 (± 5.41)	22.43 (± 4.53)	0.571
LV EF TEICHHOLZ, (%)	69.47 (± 4.96)	71.67 (± 4.20)	0.069	66.93 (± 4.16)	70.47 (± 4.80)	**0.004**
Fractional shortening (%)	37.67 (± 4.07)	39.30 (± 3.69)	0.109	36.43 (± 3.07)	39.20 (± 3.75)	**0.003**
LV mass (g)	32.36 (± 12.48)	18.48 (± 8.76)	** < 0.001**	60.76 (± 37.31)	48.75 (± 30.29)	0.176
LV mass index (g/m^2.7^)	89.72 (± 35.56)	39.93 (± 10.30)	**< 0.001**	40.97 (± 18.28)	32.70 (± 10.02)	**0.035**
Relative Wall Thickness	0.39 (± 0.10)	0.30 (± 0.05)	** < 0.001**	0.34 (± 0.08)	0.27 (± 0.04)	** < 0.001**

Statistical significance p < 0.05.

TAPSE, Tricuspid Annular Plane Systolic; LV EF, LV ejection fraction; RVDD, right ventricle diastolic diameter; LVDD, LV diastolic diameter; LVSD, LV systolic diameter; *Students t-test.

Both the two-dimensional left atrial diameter (22.32 ± 4.03 vs. 15.93 ± 2.39 mm, p < 0.001) and the left atrium volume (31.78 ± 6.35 vs. 14.25 ± 2.04 ml/m^2^, p < 0.001) were significantly higher in the liver pre-transplant group than controls. In fact, 20 (66.6%) of the 30 pre-transplant patients had Z Score >  + 2.0 when the diameter of the left atrium was analyzed.

The left ventricular mass was higher in the hepatic pre-transplant group than in the control group (32.36 ± 12.48 vs. 18.48 ± 8.76 g, p < 0.001), as well as the relative wall thickness (0.39 ± 0.10 vs. 0.30 ± 0.05). When the left ventricular mass index was evaluated, it remained significantly higher in the pre-transplantation group (89.72 ± 35.56 vs. 39.93 ± 10.30 g/m^2,7^, p < 0.001), possibly secondary to of increase the thickness of the septum and the posterior wall. Thus, 20 (66.6%) of the 30 patients had ventricular mass index > 95th percentile for age and sex.

The aortic velocity time integral (VTI) (19.42 ± 4.15 vs. 16.11 ± 2.50 cm, p = 0.001) and the pulmonary VTI (19.76 ± 3.74 vs, 16.36 ± 2.48 cm, p < 0.001) were significantly different between the hepatic pre-transplant group and the control group. Pulmonary artery systolic pressure (PASP), estimated through tricuspid reflux, although still within normal values, was higher in pre-liver transplant patients (28.90 ± 4.87 vs. 23.83 ± 3.44 mmHg, p < 0.001). In the pulsed Doppler of the mitral valve, there was a difference in the peak of the A wave velocity (80.57 ± 21.59 vs. 71.10 ± 13.77) and the E wave deceleration time (131.33 ± 24.91 vs 116.7 ± 17.25 ms, p = 0.010) (Table 3).

**Table 3:** Convetional Doppler parameters.

	Pre-Tx (n = 30) Average (SD)	Controls (n = 30) M Average (SD)	P*	Post-Tx (n = 30) Average (SD)	Controls (n = 30) Average (SD) ±	P*
Aortic VTI (cm)	19.42 (± 4.15)	16.11 (± 2.50)	**0.001**	19.66 (± 3.20)	20.04 (± 2.27)	0.604
Pulmonary VTI (cm)	19.76 (± 3.74)	16.36 (± 2.48)	** < 0.001**	18.50 (± 2.94)	19.40 (± 2.25)	0.190
RVSP (mmHg)	28.90 (± 4.87)	23.83 (± 3.44)	** < 0.001**	27.03 (± 5.56)	24.63 (± 3.23)	**0.047**
Mitral E (cm/s)	112.90 (± 27.29)	104.63 (± 12.12)	0.137	109.73 (± 16.38)	108.83 (± 14.56)	0.823
Mitral A (cm/s)	80.57 (± 21.59)	71.10 (± 13.77)	**0.048**	66.77 (± 19.28)	63.53 (± 13.49)	0.455
Mitral E/A	1.54 (± 0.49)	1.51 (± 0.28)	0.733	1.79 (± 0.47)	1.75 (± 0.32)	0.697
DT (ms)	131.33 (± 24.91)	116.67 (± 17.25)	**0.010**	139.17 (± 32.94)	137.87 (± 24.11)	0.862

Statistical significance p < 0.05.

VTI, velocity time integral; RVSP, RV *systolic pressure; DT,* deceleration time; *Students t-test.

In relation to tissue Doppler evaluation, there was a significant difference in the systolic velocity (S 'wave) of the left ventricular free wall (8.87 ± 1.57 vs. 7.23 ± 1.38 cm / s, p < 0.001), final diastolic velocity (a wave) (8.53 ± 2.32 vs. 7.03 ± 1.87 cm / s, p = 0.008) and in IVRT (39.30 ± 7.71 vs. 35, 40 ± 4.53 ms, p = 0.021), between groups pre-transplant and controls (Table 4).

**Table 4:** Tissue Doppler imaging.

	Pre-Tx (n = 30) Average (SD)	Controls (n = 30) Average (SD)	P*	Post-Tx (n = 30) Average (SD)	Controls (n = 30) Average (SD)	P*
RV e’ (cm/s)	15.77 (± 2.85)	16.27 (± 3.39)	0.539	16.50 (± 1.94)	17.43 (± 2.75)	0.134
RV a’ (cm/s)	12.43 (± 3.20)	12.83 (± 3.05)	0.622	11.53 (± 3.54)	10.43 (± 2.24)	0.157
RV e’/a’	1.34 (± 0.40)	1.31 (± 0.27)	0.700	1.54 (± 0.43)	1.76 (± 0.54)	0.081
RV S’ (cm/s)	12.67 (± 1.73)	12.37 (± 2.88)	0.627	13.37 (± 2.13)	14.47 (± 1.72)	**0.031**
RV IVRT (ms)	37.77 (± 6.45)	35.27 (± 4.03)	0.078	40.07 (± 6.24)	39.37 (± 6.17)	0.664
RV MPI	0.34 (± 0.06)	0.34 (± 0.05)	0.887	0.34 (± 0.05)	0.35 (± 0.06)	0.230
Septal e’ (cm/s)	10.83 (± 1.93)	11.70 (± 2.94)	0.183	13.27 (± 1.80)	14.40 (± 2.51)	**0.049**
Septal a’ (cm/s)	8.17 (± 1.44)	7.63 (± 1.63)	0.184	7.43 (± 2.21)	6.63 (± 1.47)	0.105
Septal e’/a’	1.38 (± 0.42)	1.61 (± 0.57)	0.091	1.94 (± 0.63)	2.30 (± 0.72)	**0.049**
Septal S’ (cm/s)	7.60 (± 0.97)	8.00 (± 2.08)	0.346	8.23 (± 1.10)	8.60 (± 1.57)	0.299
Septal IVRT (ms)	34.43 (± 8.23)	34.93 (± 4.78)	0.775	37.13 (± 7.26)	36.77 (± 5.89)	0.831
Septal MPI	0.31 (± 0.05)	0.33 (± 0.05)	0.127	0.32 (± 0.07)	0.31 (± 0.05)	0.740
Lateral e’ (cm/s)	13.37 (± 3.48)	12.10 (± 2.98)	0.135	17.50 (± 2.99)	17.97 (± 2.88)	0.541
Lateral a’ (cm/s)	8.53 (± 2.32)	7.03 (± 1.87)	**0.008**	8.83 (± 2.95)	6.70 (± 1.53)	**0.001**
Lateral e’/a’	1.65 (± 0.57)	1.86 (± 0.78)	0.254	2.19 (± 0.78)	2.82 (± 0.73)	**0.002**
Lateral S’ (cm/s)	8.87 (± 1.57)	7.23 (± 1.38)	** < 0.001**	10.10 (± 1.84)	9.87 (± 2.61)	0.691
Lateral IVRT (ms)	39.30 (± 7.71)	35.40 (± 4.53)	**0.021**	37.53 (± 4.87)	36.00 (± 4.79)	0.224
Lateral MPI	0.36 (± 0.11)	0.34 (± 0.05)	0.445	0.31 (± 0.07)	0.31 (± 0.04)	0.947
Mitral E/e’	9.55 (± 2.48)	9.18 (± 2.16)	0.542	7.31 (± 1.66)	6.88 (± 1.45)	0.291

Statistical significance p < 0.05.

IVRT, isovolumic relaxation time; MPI, Myocardial Performance Index; *Students t-test.

In the post-transplant group, there was a significant difference in the ejection fraction obtained by Simpson´s method (60.72 ± 4.31 vs. 64.07 ± 4.08%, p = 0.003), according to the fraction by Teichholz’s method (66.93 ± 4.16 vs. 70.47 ± 4.80%, p = 0.004) and with shortening fraction (36.43 ± 3.07 vs. 39.20 ± 3.75%, p = 0.003) comparing to control group (Table 2).

The left atrial diameter (25.35 ± 4.86 vs. 22.42 ± 4.38 mm, p = 0.017) as well as left atrium volume (23.24 ± 5.81 vs. 19.66 ± 4, 11 ml/m^2^, p = 0.008) were higher in the post-transplant group compared to controls. In this group also, 07 (23.3%) of the 30 patients after hepatic transplantation presented Z Score >  + 2.0 of the left atrium diameter. The difference of the diameter of the interventricular septum (6.17 ± 1.29 vs. 5.17 ± 1.15 mm, p = 0.002) and the posterior wall (5.96 ± 1.18 vs. 4.77 ± 1.22 mm, p < 0.001) between the two groups was also statistically significant higher.

When we analyzed the left ventricle indexed mass in the post-transplant group, there was a difference between the groups (40.97 ± 18.28 vs. 32.70 ± 10.02 g / m2.7, p = 0.035). Therefore, 7 (23.3%) of the 30 patients presented ventricular mass index > 95th percentile for age and sex. Relative wall thickness was significantly higher in the post-transplant group (0.34 ± 0.08 vs. 0.27 ± 0.04).

In the post-transplant group only PSAP was higher than the controls (27.03 ± 5.56 vs. 24.63 ± 3.23 mmHg, p = 0.047) (Table 3). In the post-transplant group, there was a statistically significant difference in the S' wave velocity of the right ventricle lateral wall (13.37 ± 2.13 vs. 14.47 ± 1.72 cm/s, p = 0.031), diastolic velocity (13.27 ± 1.80 vs. 14.40 ± 2.51 cm/s, p = 0.049), left ventricle lateral wall a' wave (8.83 ± 2.95 (0.94 ± 0.63 vs. 2.30 ± 0.72, p = 0.049), and in the septal relation e '/ a'. (2.19 ± 0.78 vs. 2.89 ± 0.73, p = 0.002) (Table 4).

### Evaluation through two-dimensional speckle tracking

Patients in pre-transplant group had a significant reduction in the parameters of left ventricular longitudinal myocardial deformation as shown in Table 5. The global longitudinal strain (GLS) of left ventricle was significantly lower in pre-transplant patients (−20.86 ± 2.59 vs. −22.79 ± 1.52%, p = 0.001) than controls. When analyzed separately from each window, a significant reduction of longitudinal strain was observed in the three-chamber (APLAX) (−20.38 ± 3.08 vs. −22.40 ± 1.52%, p = 0.005) and in the apical four chambers (4C) (−21.10 ± 3.05 vs. −23.57 ± 2.64%, p = 0.001). The GLS of the right ventricle also showed a significant reduction in the pre-transplant group (−23.91 ± 4.34 vs. −26.45 ± 3.65%, p = 0.017). The apex radial strain was significantly lower in pre-liver transplant patients (29.84 ± 11.12 vs. 35.14 ± 8.63, p = 0.044), as well as the radial strain at the level of the papillary muscle (25, 28 ± 8.57 vs. 31.75 ± 5.36, p = 0.001) and the overall radial strain (27.05 ± 6.92 vs. 31.54 ± 4.23, p = 0.004). The circumferential strain at the level of the papillary muscle was significantly lower in pre-liver transplant patients (−23.89 ± 4.88 vs. −26.55 ± 3.58, p = 0.019) as well as the global circumferential strain (−25.76 ± 4.42 vs. −27.84 ± 3.38, p = 0.045).

**Table 5:** Two-D Speckle Tracking parameters.

	Pre-Tx (n = 30) Average (SD)	Controls (n = 30) Average (SD)	P*	Post-Tx (n = 30) Average (SD)	Controls (n = 30) Average (SD)	P*
LV GLS (%)	−20.86 (± 2.59)	−22.79 (± 1.52)	**0.001**	−21.47 (± 2.25)	−22.89 (± 2.30)	**0.019**
APLAX (%)	−20.38 (± 3.08)	−22.40 (± 2.21)	**0.005**	−20.70 (± 3.76)	−23.33 (± 2.92)	**0.004**
4C (%)	−21.10 (± 3.05)	−23.57 (± 2.64)	**0.001**	−21.30 (± 2.90)	−23.01 (± 2.50)	**0.02**
2C (%)	−21.21 (± 3.24)	−22.43 (± 1.83)	0.078	−21.73 (± 2.30)	−22.67 (± 2.59)	0.140
RV GLS (%)	−23.91 (± 4.34)	−26.45 (± 3.65)	**0.017**	−24.59 (± 3.53)	−27.60 (± 3.35)	**0.001**
RS Apex (%)	29.84 (± 11.12)	35.14 (± 8.63)	**0.044**	29.23 (± 8.33)	31.26 (± 8.87)	0.365
RS pm (%)	25.28 (± 8.57)	31.75 (± 5.36)	**0.001**	27.85 (± 6.90)	32.14 (± 6.42)	**0.015**
RS basal (%)	26.03 (± 8.29)	27.72 (± 3.84)	0.315	25.66 (± 6.57)	29.99 (± 5.36)	**0.007**
GRS (%)	27.05 (± 6.92)	31.54 (± 4.23)	**0.004**	27.58 (± 4.90)	31.13 (± 4.89)	**0.007**
CS Apex (%)	−29.09 (± 7.10)	−31.67 (± 7.43)	0.173	−30.45 (± 8.70)	−32.70 (± 6.82)	0.270
CS pm (%)	−23.89 (± 4.88)	−26.55 (± 3.58)	**0.019**	−24.83 (± 4.20)	−26.40 (± 3.61)	0.127
CS basal (%)	−24.31 (± 4.61)	−25.29 (± 3.38)	0.350	−24.75 (± 4.26)	−26.34 (± 3.65)	0.128
GCS (%)	−25.76 (± 4.42)	−27.84 (± 3.38)	**0.045**	−26.68 (± 4.21)	−28.48 (± 3.12)	0.065

Statistical significance p < 0.05.

LV GLS, LV global longitudinal strain; APLAX, apical long-axis view; 4C, apical four *chamber view; 2C*, apical two chamber view; RV GLS, RV global longitudinal strain; RS, radial strain; Pm, papillary muscle level; GRS, global radial strain; CS: circumferential strain; GCS, global circumferential strain; *Students t-test.

Post-liver transplant patients also showed a significant reduction in the parameters of left ventricular longitudinal myocardial deformation as shown in Table 5. Left ventricular GLS was significantly lower in patients after liver transplantation (−21.47 ± 2.25 vs. −22.89 ± 2.30%, p = 0.019) comparing to controls. When analyzing each window separately, a significant reduction of longitudinal strain was observed in the APLAX (−20.70 ± 3.76 vs. −23.33 ± 2.92%, p = 0.004) and in the 4C (−21.30 ± 2.9 vs. −23.01 ± 2.50%, p = 0.02). The GLS of the right ventricle also showed a significant reduction in the post-transplant group (−24.59 ± 3.53 vs. −27.60 ± 3.35%, p = 0.001) as well as the radial strain at the level of the papillary muscle (27.85 ± 6.90 vs. 32.14 ± 6.42, p = 0.015) the radial strain at the level of the mitral valve (25.66 ± 6.57 vs. 29.99 ± 5.36, p = 0.007) and global radial strain (27.58 ± 4.90 vs. 31.13 ± 4.89, p = 0.007).

### Correlation between two-dimensional speckle-tracking variables and BNP

**Figure 2:**
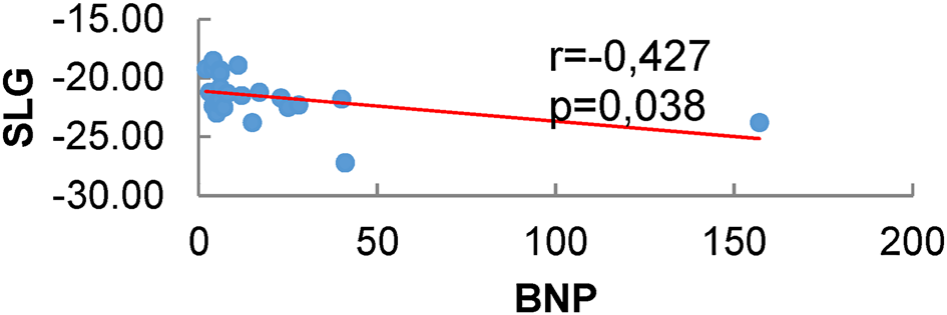
Correlation between SLG and BNP values in patients after liver transplantation.

In the post-transplantation group there was a significant correlation between BNP and GLS (p = 0.038 and r = −0.427). Therefore, the lower the BNP value, the lower the GLS value (more negative) (Fig. 2).

### Strain measures reproducibility

The reproducibility of strain measurements was analyzed in 40 randomly selected patients. The intraclass correlation of the LV global longitudinal strain was 0.92 (95% confidence interval = 0.85–0.96) for interobserver variability. Regarding intraobserver variability, the intraclass correlation of the LV global longitudinal strain was 0.93 (95% confidence interval = 0.86–0.97). The intraclass correlations of the RV longitudinal strain were 0.90 (95% confidence interval = 0.80–0.95) and 0.92 (95% confidence interval = 0.84–0.96) for inter variability and intraobserver, respectively.

## Discussion

This study is the first to identify left ventricular and right ventricular subclinical dysfunction through two-dimensional speckle tracking in pediatric patients with severe cirrhosis due to BA, listed for liver transplantation. In this group, there were no major cardiac complications in the postoperative period despite left ventricular subclinical dysfunction. It was also possible to evaluate another group of patients, already after liver transplantation, who had also left ventricular subclinical systolic dysfunction, too.

The median age of transplantation was 12.5 months. The inclusion of very young patients in the pre-liver transplant group (< five years) made the results even more impactful, making it possible to infer that even patients with a short time of cirrhosis may already have myocardial involvement, even if it is subclinical. The fact that the patients from this study were not hospitalized, and did not present clinical decompensation in the last 30 days of the study, resulted in lower PELD. However, even these patients had a lower global longitudinal strain compared to healthy controls. This raises the hypothesis that a longer exposure to a hyperdynamic circulatory stage could make this systolic dysfunction be diagnosed in the usual methods, which do not have the same sensitivity as the strain.

In this study, changes in the longitudinal, circumferential and radial strain were verified pre-liver transplantation, as well as changes in the longitudinal and radial strain post-liver transplantation. It is interesting to note that even post-liver transplantation, the values of the longitudinal strain from the right and left ventricles are lower than in control patients.

When comparing the results of the global longitudinal strain obtained in the study with the values suggested by the most recent pediatric meta-analysis and systematic review on the subject^10^, we observed that 33.3% of the patients had values below expected before liver transplantation and 36.3% post-liver transplantation. There are no cohort studies with these patients demonstrating whether these persistently reduced strain values could be related as a risk factor for cardiovascular morbidity and mortality in adults or the time after liver transplantation could cause changes in these parameters, which may affect survival in the future.

As previously described in others studies in pediatric populations with hepatic cirrhosis, conventional echocardiography showed an increase in ventricular mass index as the main structural alteration, associated with dilation of the left chambers, probably due to the hyperdynamic circulation state of these patients and difficulty in ventricular filling^1,11,12^. Grose et al. showed that final systolic and diastolic volumes increased in cirrhosis and attributed this fact to the decrease in cardiac contractility^13^.

In 2011, Desai et al. reported cardiac structural and functional alterations in infants and children with biliary atresia, listed for liver transplantation. In Desai’s report, two-dimensional echocardiography of infants with biliary atresia, listed for liver transplantation, were reviewed and compared with age and sex-matched infants without cardiac or liver disease as controls. Compared to controls, children with BA of this study had significant increases in multiple parameters, notably left ventricle wall thickness and left ventricle mass indexed, similar to the findings of our study^14^.

Studies report this change in up to 70% of pre-liver transplant patients^15^. Our data are in compliance with this study, which has found alterations in conventional echocardiography in 66.6% of the pre-liver transplant patients. There was a significant increase in the diastolic diameter of the right ventricle, also cited in other studies, especially in decompensated patients, what can be attributed to prolonged volume overload.

The LV indexed mass value was higher in patients before liver transplantation. A more recent study has chosen to calculate the LV mass through high height to 2.7 because this calculation approaches more to the lean body mass^9^. This data is important because can be used in the stratification of cardiovascular risk and may change treatment decisions^16^.

In the post-liver transplantation group, there was also a significant increase in the volume of the left atrium, out dilation of the left ventricle. Additionally, an increase in the ventricular mass index was also observed in these patients. Although it has been reported in studies that there is a reduction in the size of the left heart chambers and a reduction in left ventricular mass after transplantation, in our sample there was still a significant difference when compared to normal controls, despite of the values were still within normal limits^17^.

Although no patient in the study had left ventricular systolic dysfunction, the ejection fraction value by Simpson´s method was significantly lower in pre-liver transplant patients than in controls, even though these values were still within the normal range. This fact was not verified when analyzed the systolic function by Teichholz´s method, which method has the disadvantage of undergoing changes with ventricular geometry. Even in patients after hepatic transplantation, this alteration was maintained with statistical significancy, without an apparent clinical significance. It is speculated that perhaps this dysfunction could become clinically visible if subjected to pharmacological or physical stress tests. The systolic function is generally preserved until a very late course, so it should not be considered for the early diagnosis of cirrhotic cardiomyopathy. Our patients may have had better cardiac reserves, being able to mask the manifestations of cirrhotic cardiomyopathy more than in adults, especially those with cardiovascular risk factors.

The LV peak systolic velocity measured by TDI was not reduced in pre-liver transplant patients, however there was a higher velocity of the S’ wave in the left ventricular lateral wall, and these factors can be attributed to the hyperdynamic state that accompanies cirrhosis. The left IVRT was significantly more prolonged in pre-liver transplant patients than in controls, as well as the increase in the a' wave velocity, which may suggest a loss of left ventricular diastolic function in this subclinical stage. The underlying mechanism of diastolic dysfunction in cirrhosis is probably due to the increase in myocardial wall stiffness caused by myocardial hypertrophy, fibrosis and subendothelial edema, and subsequently resulting in high left ventricular and atrial filling pressures^18^. In patients after liver transplantation, a lower e '/ a' ratio was found in the septum and in the LV lateral wall, lower e’ septal velocity and higher a' wave velocity in the left ventricular lateral wall. This finding was described in a previous study in patients with cirrhosis due to chronic hepatitis B^19^.

The present study demonstrated a significant increase in aortic and pulmonary VTI in the hepatic pre-transplant group, probably due to the hyperdynamic circulation presented by these patients, with no statistical significance in the post-transplant group. Likewise, pulmonary artery systolic pressure, estimated through tricuspid reflux, even within normal range, was shown to be significantly increased in the pre and post-transplant groups. Pulmonary hypertension has recently been shown to develop in a substantial proportion of patients with cirrhosis. The mechanism of increased pulmonary artery pressure is not fully understood, but previous studies have suggested an increase in the levels of vasoactive substances in the pulmonary circulation and the likely toxic effect of these substances on endothelial cells. Some authors have suggested that microthrombi can migrate to the pulmonary vascular bed along portosystemic shunts and can cause an increase in vascular resistance^20^.

Pulmonary artery systolic pressure on transthoracic echocardiography may be elevated for many reasons other than portopulmonary hypertension, including venous pulmonary hypertension secondary to left ventricular dysfunction, volume overload, or increased cardiac output^1,20^.

The study of cardiac function was complemented in patients pre and post-liver transplantation using biomarkers such as BNP, who had normal values for age in all patients in this study, probably because the patients were not decompensated at the time of the examination. BNP is an excellent marker of myocardial damage and atrial distension, and although left atrial dilation was present in 66.6% of pre-liver transplant patients, the value of this marker remained within normal limits. However, in the correlation analysis, there was a significant difference, with higher BNP values associated with lower longitudinal strain values (less negative) in the group after liver transplantation. This fact indicates the need of further studies to correlate BNP with clinical cardiac alterations in these patients, maybe serving as a routine biomarker in the future. Some studies have shown that BNP was significantly higher in cirrhotic patients than in controls^17^. Other researchers reported an association between the level of BNP and pro BNP with the severity of the liver disease, or with the thickness of the posterior wall and the interventricular septum, including changes in tissue Doppler^21,22^.

Prospective studies will be needed to establish whether subclinical left ventricular and right ventricular dysfunction detected through two-dimensional speckle tracking in cirrhosis due to BA will predict adverse cardiovascular events in the future. In addition, the inclusion of two-dimensional speckle tracking in the routine echocardiographic evaluation of patients listed for liver transplantation may help to determine if any early therapeutic intervention could modify the cardiovascular prognosis.

### Limitation

As a limitation of the present research, this was a relatively small observational study and conducted in a single call center, which may hinder the generalization of our conclusions for larger populations. In addition, patients evaluated before liver transplantation are not part of the same cohort as patients post-liver transplantation, which makes it difficult to infer whether the changes found in the post-transplantation would be due to changes already established before the transplant or acquired (cumulative effect) during treatment.

## Conclusion

The present study demonstrated the presence of a significant reduction in the values of the global longitudinal strain of the left and right ventricles in the group before and after liver transplantation. It also demonstrated a reduction in the radial and circumferential strain in the pre-transplant group and a reduction in the radial strain in the post-liver transplant, through the assessment of myocardial deformation using the two-dimensional speckle tracking technique. Lower BNP levels were associated with lower longitudinal strain values in the group after liver transplantation.

## Data Availability

The datasets generated during and/or analysed during the current study are available from the corresponding author on reasonable request.

## Abbreviations

CHD: Congenital heart diseases
BA: Biliary atresia
LT: Liver transplant
CVD: Cardiovascular disease
US: Ultrasound
Tx: Transplant
BNP: Brain natriuretic peptide
EF: Ejection fraction
2D: Two dimensions
PELD: Pediatric end-stage liver disease
TAPSE: Tricuspid annular plane systolic
RVDD: Right ventricle diastolic diameter
RWT: Relative wall thickness
LVSD: Left ventricular systolic diameter
LVDD: Left ventricular diastolic diameter
LVEF: Left ventricular ejection fraction
LVFS: Left ventricular fractional shortening
LAD: Left atrial diameter
TDI: Tissue doppler imaging
VTI: Time-velocity integral
RVSP: RV systolic pressure
DT: Deceleration time
IVRT: Isovolumic relaxation time
MPI: Myocardial performance index
PASP: Pulmonary artery systolic pressure
LV GLS: LV global longitudinal strain
APLAX: Apical long-axis view
4C: Apical four chamber view
2C: Apical two chamber view
RV GLS: RV global longitudinal strain
RS: Radial strain
Pm: Papillary muscle level
GRS: Global radial strain
CS: Circumferential strain
GCS: Global circumferential strain
